# Endovascular Treatment Versus Vein Bypass of Infrainguinal Peripheral Artery Disease: A Systematic Review and Meta-Analysis of Randomized Controlled Trials

**DOI:** 10.3390/jcm15010002

**Published:** 2025-12-19

**Authors:** Yuhan Qi, Yu Yang, Chengxin Weng, Jichun Zhao, Jiarong Wang, Ding Yuan, Tiehao Wang

**Affiliations:** 1 Division of Vascular Surgery, Department of General Surgery, West China Hospital, Sichuan University, 37 Guo Xue Alley, Chengdu 610041, China; 2 West China School of Medicine, Sichuan University, Chengdu 610041, China

**Keywords:** endovascular treatment, vein bypass, infrainguinal peripheral artery disease, meta-analysis

## Abstract

**Objectives:** Guidelines widely recommend endovascular therapy (ET) for infrainguinal peripheral artery diseases (PAD) despite the lack of adequately powered data. This meta-analysis aimed to directly compare the clinical safety and efficacy of ET versus vein bypass (VBP) in patients with infrainguinal PAD from available randomized controlled trials (RCTs). **Methods:** We conducted a systematic literature search of MEDLINE, Embase, and the Cochrane databases from inception until 21 July 2023 for RCTs comparing ET and VBP. Treatment effects were expressed as odds ratios (OR) with 95% confidence intervals (CIs), pooled using the Mantel–Haenszel method. Study quality was assessed via the Cochrane Risk of Bias tool. **Results:** From 2210 identified studies, four low-risk-of-bias RCTs were included. Pooled analysis demonstrated that ET was associated with significantly higher risks of reintervention (OR = 4.69, 3.69–6.04), major reintervention (OR = 2.80, 1.96–4.00), and any reintervention (OR = 1.92, 1.44–2.56) compared to VBP. ET also showed a lower rate of index procedure technical success (OR = 0.13, 0.07–0.25) and site infection (OR = 0.05, 0.01–0.25). However, no significant differences were observed between the two strategies regarding 30-day mortality (OR = 0.66, 0.34–1.29), all-cause mortality (HR = 1.05, 0.90–1.22), index limb amputation (OR = 1.29, 0.90–1.86), MACE (OR = 1.20, 0.94–1.54), or bleeding events (OR = 0.86, 0.30–2.50). **Conclusions:** This analysis of RCT data still supports VBP, which retained certain advantages over ET for patients with infrainguinal peripheral artery disease in terms of both efficacy and safety. In cases where a suitable vein is available, vein bypass should be considered as the primary treatment option.

## 1. Introduction

Peripheral artery disease (PAD) is a prevalent condition affecting more than 200 million people worldwide. Chronic limb-threatening ischemia (CLTI) presents as chest pain, ischemic ulcers, or gangrene in the foot and occurs in up to 11% of PAD cases [1,2]. In addition to the severe health consequences of CLTI, there is a significant economic burden, with annual costs in the United States estimated at around $12 billion [3].

Over the past decades, bypass surgery (BSx) and endovascular treatment (ET) have been introduced as the mainstream treatments for PAD. However, the choice of treatment for lesions in PAD varies, and the optimal treatment approach has been a subject of ongoing controversy. In recent years, due to the increasing development of endovascular device, the Trans-Atlantic Inter-Society Consensus (TASC II), European Society of Cardiology (ESC), and European Society for Vascular Surgery (ESVS) endorse an “endovascular-first” approach for lesions shorter than 25 cm in PAD [4,5,6]. A previous meta-analysis also showed that ET could be an effective and safe alternative to BSx in patients with symptomatic femoropopliteal disease [7]. However, previous meta-analyses and guidelines have often grouped together vein bypass and prosthetic grafts, which has not definitively elucidated the advantages and disadvantages of endovascular therapy and vein bypass for infrainguinal chronic limb-threatening ischemia. Consequently, this may have somewhat influenced the advantages of vein bypass surgery in lower-limb arterial disease, leading to heterogeneity in decision-making regarding surgical bypass procedures.

Additionally, BASIL-1 and BASIL-2 trials showed that an endovascular treatment strategy offered better amputation-free survival but was not associated with a higher risk of major adverse limb events than vein bypass surgery [8,9,10]. However, the BEST-CLI trial, a randomized controlled trial (RCT) aimed at determining whether ET was superior to vein bypass in patients with infrainguinal PAD, demonstrated that the use of the great saphenous vein for surgical revascularization significantly reduced the risk of major adverse limb events compared to endovascular therapy [11].

Given the persistent uncertainty in selecting the optimal revascularization strategy, we designed this first meta-analysis of RCT to directly evaluate safety and efficacy of vein bypass versus endovascular therapy in infrainguinal peripheral artery disease.

## 2. Methods

### 2.1. Search Strategy and Selection Criteria

This study reported in accordance with PRISMA (Preferred Reporting Items for Systematic Reviews and Meta-Analyses) and AMSTAR (Assessing the Methodological Quality of Systematic Reviews) Guidelines [12,13]. Systematic searches of published studies were performed in MEDLINE, EMBASE, and Cochrane Library from inception through to 21 July 2025 without language restrictions. The keywords for the search are listed as follows: “vein bypass”, “endovascular treatment”, and “peripheral artery disease”. Gray literature was not included in the search strategy. The full search strategy is detailed in Appendix S1. Two independent (YQ, YY) reviewers screened titles, abstracts, and subsequently full-text articles against pre-defined inclusion criteria: (1) clinical studies reporting outcomes of patients with chronic limb-threatening ischemia; (2) RCTs comparing treatment with vein bypass surgery versus endovascular treatment with or without stent; and (3) crossover trials were excluded in our study. Discrepancies were resolved through discussion or consultation with a third reviewer (DY, TW).

### 2.2. Data Analysis

Data were extracted independently by two reviewers (YQ, YY) using a standardized form. Any disagreements were adjudicated by a third reviewer (DY, TW). It was subsequently verified that no duplicate publications existed among the selected trials.

Collected baseline characteristics included study and year, clinical status of patients, study design, time period, study centers, simple size, age, sex, ET procedure, and VBP procedure. The efficacy outcomes of interest were reintervention (defined as repeat procedure of the same type), major reintervention (defined as new bypass, graft revision, thrombectomy, or thrombolysis), any reintervention, amputation of the index limb (comprising major and minor amputations), and technical success (defined as achieving a patent bypass graft at the completion of the procedure, or attaining a residual stenosis level below 50% within the target lesion, along with at least one outflow artery or successfully treated outflow below the knee). The safety outcomes of interest were 30-day mortality, all-cause mortality, bleeding events (classified as major or minor per study definitions), major adverse cardiovascular events (MACE), (MACE is defined as a composite of death, myocardial infarction, transient ischemic attack, and stroke within 30 days), and site infection of the index limb.

The risk of bias for each RCT was assessed independently by two reviewers (YQ, JW) using the Cochrane RoB 2 tool (RoB 2) [14]. Sensitivity analysis was performed by sequentially excluding individual studies to explore the stability of the findings. The number of patients needed to treat (NNT) or harm (NNH) were calculated based on the absolute risk differences.

Statistical analyses were conducted using Review Manager Version 5.3 (The Nordic Cochrane Centre, København, Denmark) following the methods described in the Cochrane Handbook for Systematic Reviews of Interventions (version 6.2). For dichotomous data, odds ratios (OR) with 95% CIs were calculated using the Mantel–Haenszel method. For time-to-event data, a generic inverse-variance method was used to summarize hazard ratios (HR) from results of Cox proportional analysis.

Regression models and HR were estimated according to Tierney methods [15]. Heterogeneity was quantified using the I^2^ statistic, where I^2^ > 75% indicated high heterogeneity [16].

## 3. Results

The initial search yielded 2237 records. After duplicate removal and screening, the literature selection generated five articles [10,11,17,18,19], which includes four RCTs [10,11,17,18] that were deemed suitable for inclusion in the quantitative analysis. The PRISMA flow diagram is presented in Figure S1, and the PRISMA checklist is available in Table S1. Four studies reported outcomes of reintervention, major reintervention, any reintervention, 30-day mortality, amputation of the index limb, MACE, and technical success of index procedure. Key study characteristics are summarized in Table 1. The assessment of bias risk using the ROB 2 tool for RCTs revealed a low risk of bias across most domains for all included trials (Figure S2).

### 3.1. Efficacy Outcomes

**Figure 1:**
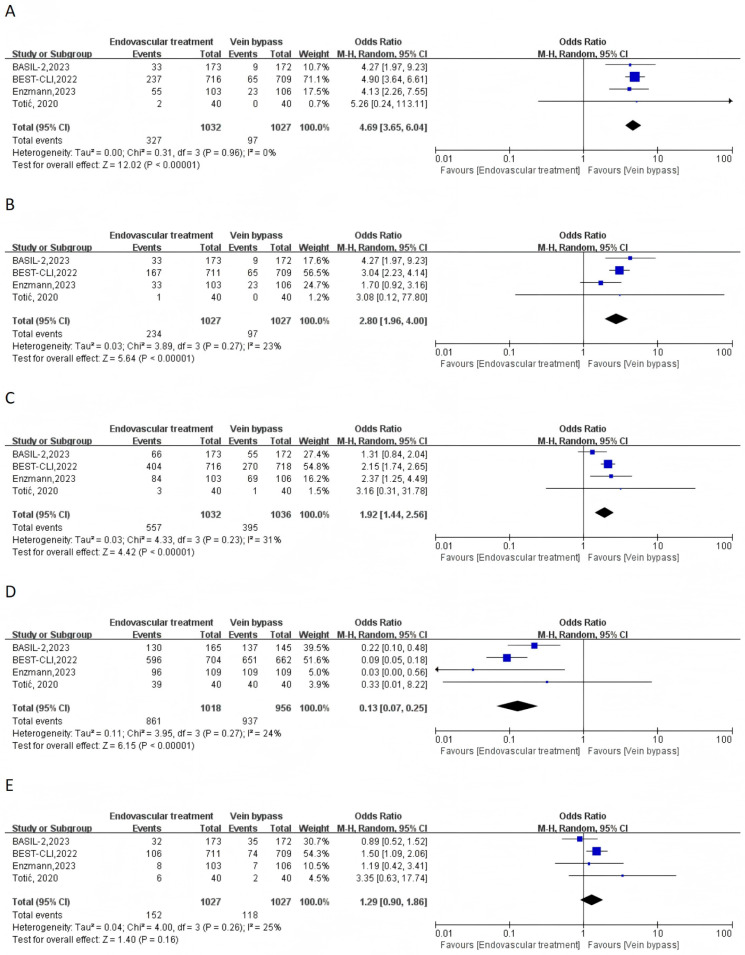
Forest plots of endovascular treatment versus vein bypass for the efficacy outcomes: (**A**) reintervention; (**B**) major reintervention; (**C**) any reintervention; (**D**) technical success of index procedure; (**E**) amputation. CI = confidence intervals; M-H = Mantel–Haenszel; IV = inverse variance [10,11,17,18].

Four RCTs were included for the primary outcomes of reintervention event, major reintervention event, and any reintervention. The pooled estimate incidence of reintervention event was 31.69% (327/1032) and 9.44% (97/1027) in the ET and VBP groups. Compared to the VBP group, ET was associated with markedly increased odds of reintervention (OR = 4.69, 95% CI 3.69–6.04, I^2^ = 0%), major reintervention (OR = 2.80, 95% CI 1.96–4.00, I^2^ = 23%), and any reintervention (OR = 1.92, 95% CI 1.44–2.56, I^2^ = 31%) (Figure 1A–C). Additionally, the pooled estimate incidence of technical success was 84.58% (861/1018) and 98.01% (937/956) in the ET and VBP groups. Compared to the VBP group, ET exhibited a lower rate of technical success of the index procedure in patients with PAD (OR = 0.13, 95% CI 0.07–0.25, I^2^ = 24%) (Figure 1D). The pooled estimate incidence of amputation of the index limb was 14.80% (152/1027) and 11.49% (118/1027) in the ET and VBP groups. No significant difference was found in the risk of index limb amputation between ET and VBP (OR = 1.29, 95% CI 0.90–1.86, I^2^ = 25%) (Figure 1E).

### 3.2. Safety Outcomes

**Figure 2:**
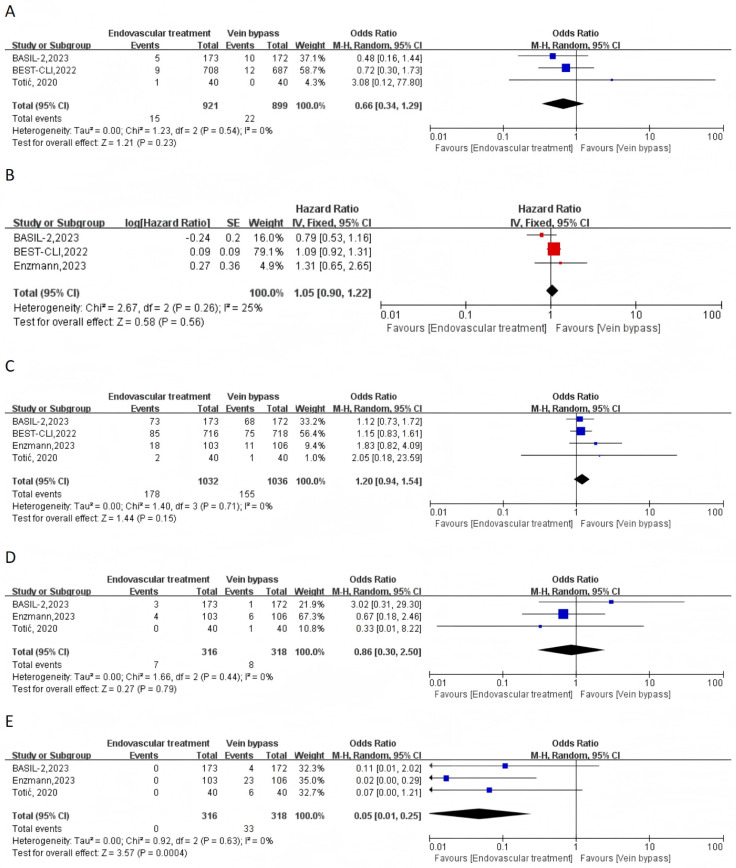
Forest plots of endovascular treatment versus vein bypass for the safety outcomes: (**A**) 30-day mortality; (**B**) all-cause mortality; (**C**) MACE; (**D**) bleeding; (**E**) site infection). CI = confidence intervals; M-H = Mantel–Haenszel; IV = inverse variance [10,11,17,18].

In terms of safety outcomes, compared to the VBP group, ET was not associated with a reduction of 30-day mortality rate (OR = 0.66, 95% CI 0.34–1.29, I^2^ = 0%) and all-cause mortality rate (HR = 1.05, 95% CI 0.90–1.22, I^2^ = 25%) in patients with PAD (Figure 2A,B). ET was also not associated with a reduced risk of MACE (OR = 1.20, 95% CI 0.94–1.54, I^2^ = 0%) or the risk of bleeding (OR = 0.86, 95% CI 0.30–2.50, I^2^ = 0%) in patients with PAD (Figure 2C,D). Compared to the VBP group, ET was associated with a lower risk of site infection of the index limb in patients with PAD (OR = 0.05, 95% CI 0.01–0.25, I^2^ = 0%) (Figure 2E).

### 3.3. Sensitivity Analyses and NNT/NNH

Sensitivity analyses, including the exclusion of any single study (Table S2) and a direct comparison between ET and bypass surgery using the great saphenous vein (Figures S4 and S5), confirmed the robustness of the primary outcomes, as neither approach substantially altered the pooled estimates. The calculated NNT to prevent one reintervention with VBP was 4, for major reintervention was 7, for any reintervention was 6, and for technical success of index limb was 8. Conversely, the NNH for causing one additional site infection with VBP was 10 (Figure S3).

## 4. Discussion

The key findings of the present analysis of RCT that investigate the efficacy and safety of endovascular therapy compared to vein grafts bypass in patients with infrainguinal peripheral artery disease are the following.

Vein bypass demonstrated a lower risk of reintervention, major reintervention, and any reintervention, as well as a higher technical success of index procedure, although it was associated with an increased risk of site infection. No significant differences were observed between endovascular therapy and vein bypass regarding MACE, all-cause mortality, 30-day mortality, amputation of the index limb, and bleeding.

Over the past decades, bypass surgery has been widely recognized as the gold standard in the care of patients with PAD, particularly those with complex lesions [6,20,21,22]. What is noteworthy is that with increasing development of endovascular devices, endovascular therapy may indicate comparable primary patency rates to bypass surgery [23,24]. For instance, a two-year RCT comparing nitinol stent implantation with bypass surgery for long femoropopliteal lesions showed similar freedom from target lesion revascularization rates [19]. Another multicenter RCT comparing femoropopliteal bypass with heparin-bonded expanded polytetrafluoroethylene endografts also demonstrated no difference in target lesion revascularization [23]. Additionally, a recent meta-analysis showed that bypass surgery was not associated with decreased risk of reintervention of target limbs [7]. It is worth noting that when drawing conclusions about reintervention between endovascular therapy and bypass surgery, there was insufficient distinction made between venous bypass and prosthetic bypass, which may introduce certain biases when interpreting the results. Studies have shown that patients who undergo venous bypass have better patency rates compared to those who undergo prosthetic bypass [25,26]. Furthermore, in previous trials, where venous grafting and prosthetic grafting were combined as the same study group, bypass surgery did not demonstrate significant benefits over endovascular treatment in reducing reinterventions [23,27]. Prosthetic grafting may partially diminish the advantages of venous grafting in terms of reinterventions. This may be attributed to the similar primary patency and secondary patency observed between two groups [28,29]. In our present meta-analysis, data collated from recent RCTs further demonstrated that patients undergoing vein bypass exhibit a lower risk of postoperative reintervention, major reintervention, and any intervention compared to those undergoing endovascular therapy. This may be attributed to the higher patency rates observed in patients receiving venous bypass [17,30]. However, it should be noted that differences exist in the patient inclusion criteria across the literature we included. But all patients had chronic limb-threatening ischemia (CLTI) and required surgical revascularization. Moreover, most pooled results showed low heterogeneity (I^2^). Therefore, such differences were deemed negligible. Nevertheless, further subgroup analyses, such as by anatomical location and risk stratification, are warranted.

Our findings indicate that patients undergoing vein bypass have a higher technical success rate compared to those receiving endovascular therapy. This could be related to the complexity of peripheral vascular disease below the inguinal region, including the presence of multi-segment vessel calcification, calcification accompanied by thrombosis, and vessel tortuosity at articulation sites. In endovascular patients, therapy devices pass through the diseased segments, increasing surgical complexity, particularly in complex lesions, which may lead to acute thrombus formation and dissections, reducing the success rate of the procedure. However, in patients undergoing vein bypass, the venous conduit bypasses the diseased segment, avoiding complications associated with the lesion vessels. Similar results have also been corroborated in studies involving prosthetic bypass in the treatment of peripheral artery disease [7,31].

The safety of endovascular therapy has been well established in treating diseases of the thoracic aorta, abdominal aorta, and iliac arteries [32,33,34]. However, the safety of endovascular treatment seems to be less evident in peripheral arterial disease in the lower extremities. The results of our study demonstrated that patients undergoing venous grafting did not experience an increased overall risk of mortality or an increased risk of mortality within 30 days postoperatively (30-day mortality, generally referred to as procedure-related death), which is consistent with a previous indirect comparison by Bayesian network meta-analysis of drug-coated devices versus vein bypass in femoropopliteal arterial occlusive disease [35]. Additionally, vein bypass did not increase the risk of amputation of the index limb, MACE or bleeding. In other words, patients with PAD who were considered suitable candidates for vein bypass surgery demonstrated comparable peri-procedural complications regardless of whether they underwent VBP or ET. This finding suggests that the risk of peri-procedure complications should not be a deterrent when deciding on the appropriate revascularization strategy for PAD patients. Though just like our previous research, venous bypass increases the risk of postoperative infections [30]; prophylactic use of antibiotics can significantly reduce the risk of postoperative infections in patients undergoing lower limb revascularization surgery [36].

### Limitation

In this study, despite being the first meta-analysis of high-quality RCTs assessing the safety and efficacy of endovascular therapy compared to vein bypass in peripheral arterial disease, there are some limitations that need to be considered. Firstly, the specific techniques used in endovascular therapy were not well defined in this study, which may introduce certain biases. However, all studies compared clinical outcomes between a best endovascular treatment strategy and surgical venous bypass; therefore, pooling these studies is reasonable. Secondly, with the advent of mechanical thrombectomy techniques, the combination of mechanical thrombectomy devices with drug-coated technologies has become increasingly accepted. However, high-quality randomized controlled trials directly comparing these approaches remain lacking, which may represent an important focus for future research. Lastly, the follow-up duration varied from 1 to 4 years among the included studies, potentially introducing some interpretation biases in the results. Long-term follow-up results are still pending in the future.

## 5. Conclusions

In patients with infrainguinal peripheral artery disease, compared to endovascular therapy, vein bypass was associated with a lower risk of reintervention and a higher technical success rate of the index procedure, without an associated higher risk of mortality, MACE, amputation, and bleeding. This analysis of RCT data continues to support the efficacy and safety of vein bypass, which retained certain advantages over endovascular therapy in this patient population. When a suitable vein is available, vein bypass should be prioritized as the primary treatment option.

## Figures and Tables

**Table 1:** General characteristics of the four randomized controlled trials on ET versus VBP in PAD patients.

Study, Year	BASIL-2, 2023 [10]	BEST-CLI, 2022 [11]	Enzmann, 2023 [17]	Totić, 2020 [18]
Clinical status	infrapopliteal CLTI, with or without an additional more proximal infra-inguinal	CLTI	femoropopliteal TASC II C and D lesions	femoropopliteal TASC II B and C lesions
Design	open-label	open-label	open-label	open-label
Time period (year)	2014 to 2020	2014 to 2021	2016 to 2020	2012 to 2016
Location	41 centers in the UK, Sweden, and Denmark	150 centers in the USA	One center in the Austria	One center in Bosnia and Herzegovina
Sample size (ET/VBP)	173/172	716/718	103/106	40/40
Median or mean age-y (ET/VBP)	72.5/72.4	67.0/66.9	69.3/68.5	67/65
Male % (ET/VBP)	82%/81%	71.1/72%	67%/74%	60%/85%
Mean BMI (ET/VBP) (kg/m^2^)	26.8/27.1	28.3/28.2	26.5/26.6	NA
Diabetes % (ET/VBP)	69%/68%	71.6%/72.1%	35%/38%	57.5%/50%
Hypertension % (ET/VBP)	75%/74%	86.8%/87.1%	86%/83%	85%/72.5%
CAD % (ET/VBP)	13%/24%	44.4%/42.3%	47%/29%	12.5%/15%
Hemodialysis % (ET/VBP)	6%/3%	11.8%/9.4%	3%/3%	0%/0%
Smoking % (ET/VBP)	53%/44%	34.4%/37.1%	35%/37%	85%/90%
ET procedure	ET *	ET *	angioplasty and nitinol stents	angioplasty with or without stenting
VBP procedure	any vein bypass	saphenous vein bypass	any vein bypass	any vein bypass

CLTI: chronic limb-threatening ischemia; ET: endovascular treatment; VBP: vein bypass; TASC: Trans-Atlantic Intersociety Consensus. * ET consists of atherectomy, angioplasty, drug-coated balloon angioplasty, bare metal stents, drug-eluting stents, and stent-grafts. CAD: coronary artery disease.

## Data Availability

The dataset generated and analyzed is available from the corresponding author upon reasonable request.

## Supplementary Materials

The following supporting information can be downloaded at: https://www.mdpi.com/article/10.3390/jcm15010002/s1, Figure S1. PRISMA flow diagram. Figure S2. Risk of bias assessment of included trials. Figure S3. Number needed to treat (NNT) and number needed to harm (NNH). Figure S4. Forest plots of endovascular treatment vs. great saphenous vein only bypass for the efficacy outcome (A: reintervention; B: major reintervention; C: any reintervention; D: technical success of index procedure; E: amputation). CI = confidence intervals; M-H = Mantel-Haenszel; IV = inverse variance. Figure S5. Forest plots of endovascular treatment vs. great saphenous vein only bypass for the safety outcomes (A: 30-day mortality; B: all-cause mortality; C: MACE; D: bleeding; E: site infection). CI = confidence intervals; M-H = Mantel-Haenszel; IV = inverse variance. Table S1. PRISMA checklist. Table S2. Sensitivity analysis [37]. Appendix S1. Detailed search strategies.
