# Supplementary material for: Endovascular Treatment Versus Vein Bypass of Infrainguinal Peripheral Artery Disease: A Systematic Review and Meta-Analysis of Randomized Controlled Trials
Source: J Clin Med. 2025 Dec 19;15(1):2. doi: 10.3390/jcm15010002 (PMC12786405; doi:10.3390/jcm15010002)
Supplement: Supplementary file 1 [file jcm-15-00002-s001.zip › Appendix S1. Search strategies.pdf]

Appendix 1. Detailed search strategies

Ovid MEDLINE(R) and Epub Ahead of Print, In-Process, In-Data-Review & Other Non-Indexed Citations, Daily and Versions <1946 to July 21, 2025>

1 exp Peripheral Arterial Disease/ 18802
2 exp Femoral Artery/ 30772
3 exp Popliteal Artery/ 10398
4 infrapopliteal.mp. 1316
5 infrainguinal*.mp. 2310
6 popliteal*.mp. 22407
7 infrapopliteal*.mp. 1317
8 exp Endovascular Procedures/ 146132
9 exp Stents/ 89888
10 endovascular surgery.mp. 1372
11 endovascular treatment.mp. 17067
12 (hybrid or endovascular or stent* or balloon).mp. [mp=title, book title, abstract, original title, name of substance word, subject heading word, floating sub-heading word, keyword heading word, organism supplementary concept word, protocol supplementary concept word, rare disease supplementary concept word, unique identifier, synonyms, population supplementary concept word, anatomy supplementary concept word] 488825
13 8 or 9 or 10 or 11 or 12 535594
14 Vascular Surgical Procedures/ 33720
15 femoropopliteal bypass.mp. 790
16 vein bypass.mp. 2131
17 Saphenous vein bypass.mp. 1110
18 exp Saphenous Vein/ 16404
19 14 or 15 or 16 or 17 or 18 50524
20 13 and 19 9126
21 1 or 2 or 3 or 4 or 5 or 6 or 7 62907
22 20 and 21 1527
23 exp Peripheral Arterial Disease/ 18802
24 exp Femoral Artery/ 30772
25 exp Popliteal Artery/ 10398
26 femoral artery.mp. 41260
27 femoropopliteal.mp. 4236
28 popliteal artery.mp. 12106
29 infrapopliteal.mp. 1316
30 femor*.mp. 204343
31 infrainguinal*.mp. 2310
32 popliteal*.mp. 22407
33 infrapopliteal*.mp. 1317
34 23 or 24 or 25 or 26 or 27 or 28 or 29 or 30 or 31 or 32 or 33 231491
35 exp Endovascular Procedures/ 146132
36 exp Stents/ 89888
37 endovascular surgery.mp. 1372
38 endovascular treatment.mp. 17067
39 (hybrid or endovascular or stent* or balloon).mp. [mp=title, book title, abstract, original title, name of substance word, subject heading word, floating sub-heading word, keyword heading

word, organism supplementary concept word, protocol supplementary concept word, rare disease supplementary concept word, unique identifier, synonyms, population supplementary concept word, anatomy supplementary concept word] 488825
40 35 or 36 or 37 or 38 or 39 535594
41 Vascular Surgical Procedures/ 33720
42 femoropopliteal bypass.mp. 790
43 vein bypass.mp. 2131
44 Saphenous vein bypass.mp. 1110
45 exp Saphenous Vein/ 16404
46 41 or 42 or 43 or 44 or 45 50524
47 40 and 46 9126
48 34 and 47 1906

Embase <1974 to 2025 July 20>

1 exp peripheral arterial disease/ 27497
2 exp peripheral arterial disease/ 27497
3 peripheral arterial disease.mp. 20108
4 infrapopliteal.mp. 1969
5 infrainguinal*.mp. 3271
6 popliteal*.mp. 27520
7 exp femoral artery/ 37123
8 exp popliteal artery/ 12796
9 1 or 2 or 3 or 4 or 5 or 6 or 7 or 8 101261
10 exp endovascular surgery/ 51481
11 exp stent/ 216096
12 (endovascular or stent* or balloon).mp. [mp=title, abstract, heading word, drug trade name, original title, device manufacturer, drug manufacturer, device trade name, keyword heading word, floating subheading word, candidate term word] 446172
13 10 or 11 or 12 447313
14 exp vein bypass/ 2140
15 exp saphenous vein graft/ 8204
16 14 or 15 10214
17 13 and 16 3129
18 9 and 17 421

Cochrane

cochrane central register of controlled trials for randomized controlled trials:
Peripheral Arterial Disease and endovascular **and** vein bypass 20
