# Supplementary material for: Endovascular Treatment Versus Vein Bypass of Infrainguinal Peripheral Artery Disease: A Systematic Review and Meta-Analysis of Randomized Controlled Trials
Source: J Clin Med. 2025 Dec 19;15(1):2. doi: 10.3390/jcm15010002 (PMC12786405; doi:10.3390/jcm15010002)
Supplement: Supplementary file 1 [file jcm-15-00002-s001.zip › Figures S4 and S5.pdf]

A

Study or Subgroup	Endovascular treatment Events	Endovascular treatment Total	Vein bypass Events	Vein bypass Total	Weight	Odds Ratio M-H, Random, 95% CI
BEST-CLI,2022	237	716	65	709	79.7%	4.90 [3.64, 6.61]
Enzmann,2023	55	103	23	106	19.6%	4.13 [2.26, 7.55]
Totić ,2020	2	40	0	40	0.8%	5.26 [0.24, 113.11]
**Total (95% CI)**		**859**		**855**	**100.0%**	**4.74 [3.63, 6.19]**
Total events	294		88			

Heterogeneity: Tau² = 0.00; Chi² = 0.25, df = 2 (P = 0.88); I² = 0%
Test for overall effect: Z = 11.44 (P < 0.00001)

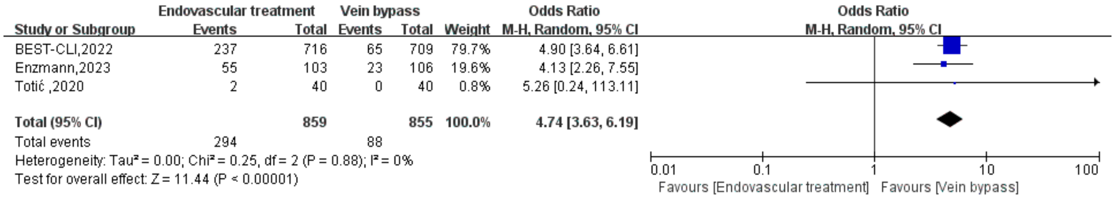

B

Study or Subgroup	Endovascular treatment Events	Endovascular treatment Total	Vein bypass Events	Vein bypass Total	Weight	Odds Ratio M-H, Random, 95% CI
BEST-CLI,2022	167	711	65	709	66.8%	3.04 [2.23, 4.14]
Enzmann,2023	33	103	23	106	31.6%	1.70 [0.92, 3.16]
Totić ,2020	1	40	0	40	1.6%	3.08 [0.12, 77.80]
**Total (95% CI)**		**854**		**855**	**100.0%**	**2.53 [1.67, 3.84]**
Total events	201		88			

Heterogeneity: Tau² = 0.04; Chi² = 2.71, df = 2 (P = 0.26); I² = 26%
Test for overall effect: Z = 4.38 (P < 0.0001)

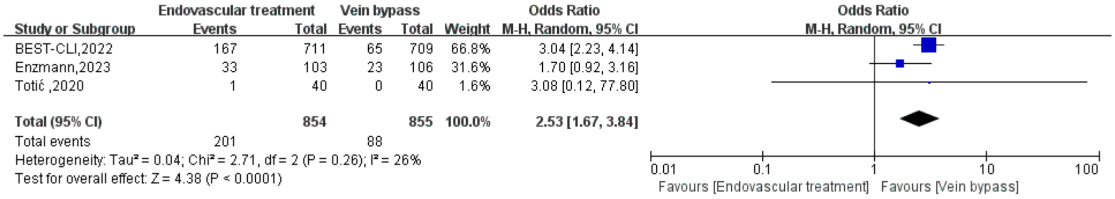

C

Study or Subgroup	Endovascular treatment Events	Endovascular treatment Total	Vein bypass Events	Vein bypass Total	Weight	Odds Ratio M-H, Random, 95% CI
BEST-CLI,2022	404	716	270	718	89.5%	2.15 [1.74, 2.65]
Enzmann,2023	84	103	69	106	9.8%	2.37 [1.25, 4.49]
Totić ,2020	3	40	1	40	0.7%	3.16 [0.31, 31.78]
**Total (95% CI)**		**859**		**864**	**100.0%**	**2.18 [1.78, 2.66]**
Total events	491		340			

Heterogeneity: Tau² = 0.00; Chi² = 0.18, df = 2 (P = 0.91); I² = 0%
Test for overall effect: Z = 7.63 (P < 0.00001)

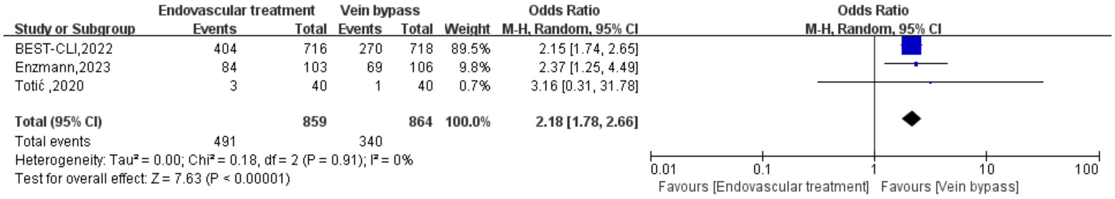

D

Study or Subgroup	Endovascular treatment Events	Endovascular treatment Total	Vein bypass Events	Vein bypass Total	Weight	Odds Ratio M-H, Random, 95% CI
BEST-CLI,2022	596	704	651	718	99.0%	0.57 [0.41, 0.79]
Enzmann,2023	96	109	109	106		Not estimable
Totić ,2020	39	40	40	40	1.0%	0.33 [0.01, 8.22]
**Total (95% CI)**		**853**		**864**	**100.0%**	**0.56 [0.41, 0.78]**
Total events	731		800			

Heterogeneity: Tau² = 0.00; Chi² = 0.11, df = 1 (P = 0.74); I² = 0%
Test for overall effect: Z = 3.47 (P = 0.0005)

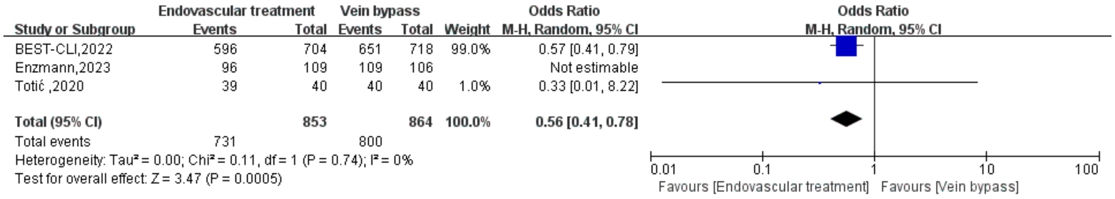

E

Study or Subgroup	Endovascular treatment Events	Endovascular treatment Total	Vein bypass Events	Vein bypass Total	Weight	Odds Ratio M-H, Random, 95% CI
BEST-CLI,2022	106	711	74	709	88.7%	1.50 [1.09, 2.06]
Enzmann,2023	8	103	7	106	8.1%	1.19 [0.42, 3.41]
Totić ,2020	6	40	2	40	3.2%	3.35 [0.63, 17.74]
**Total (95% CI)**		**854**		**855**	**100.0%**	**1.51 [1.12, 2.04]**
Total events	120		83			

Heterogeneity: Tau² = 0.00; Chi² = 1.08, df = 2 (P = 0.58); I² = 0%
Test for overall effect: Z = 2.72 (P = 0.006)

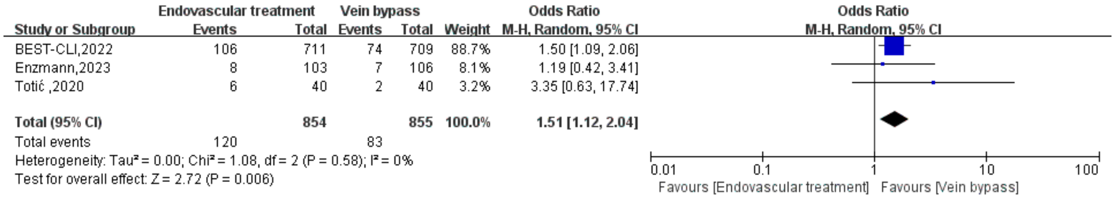

Figure S4. Forest plots of endovascular treatment vs. great saphenous vein only bypass for the efficacy outcome (A:reintervention; B: major reintervention; C: any reintervention; D: technical success of index procedure; E: amputation). CI = confidence intervals; M-H = Mantel-Haenszel; IV = inverse variance.

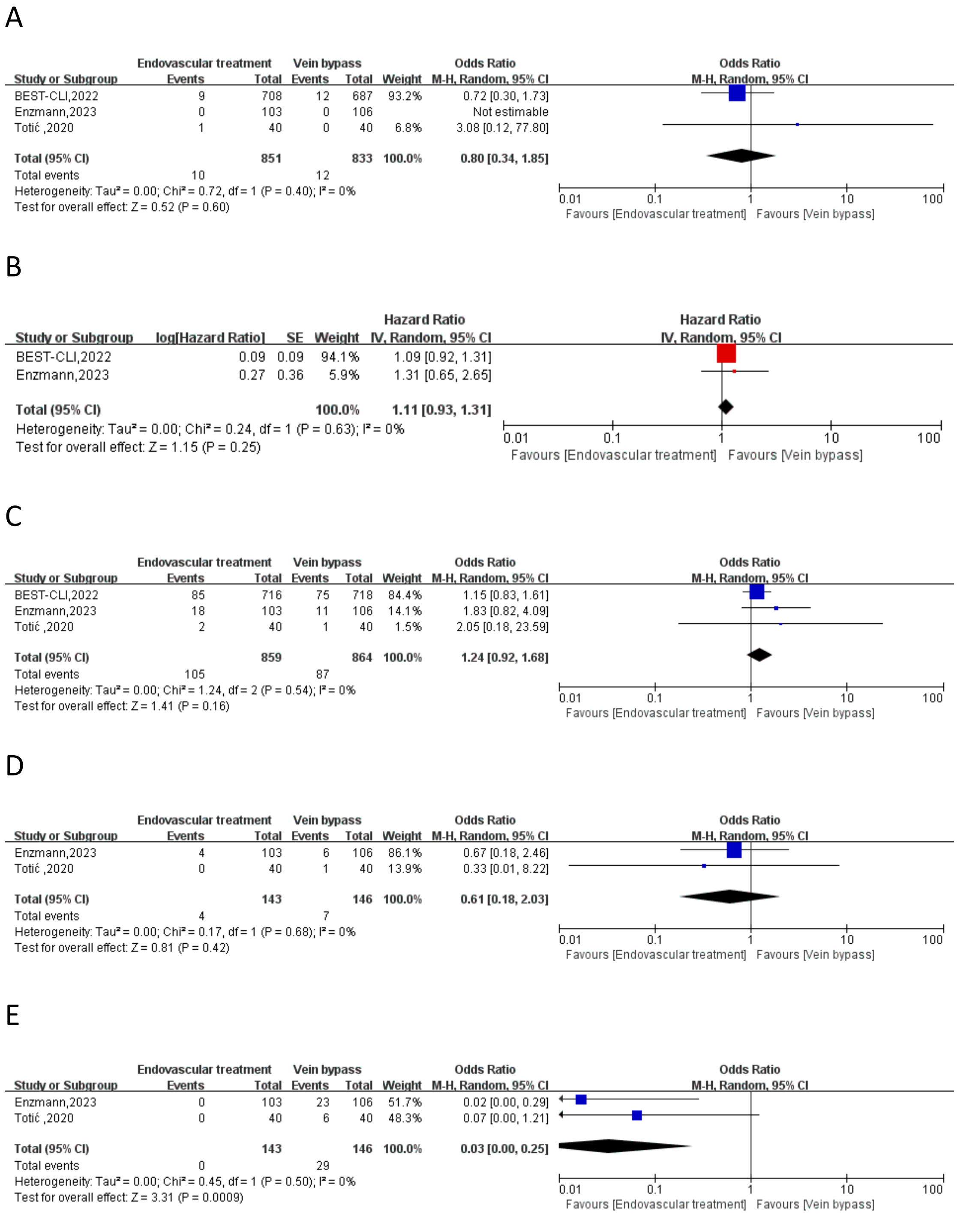

Figure S5. Forest plots of endovascular treatment vs. great saphenous vein only bypass for the safety outcomes (A: 30-day mortality; B: all-cause mortality; C: MACE; D: bleeding; E: site infection). CI = confidence intervals; M-H = Mantel-Haenszel; IV = inverse variance.
