# Supplementary material for: Endovascular Treatment Versus Vein Bypass of Infrainguinal Peripheral Artery Disease: A Systematic Review and Meta-Analysis of Randomized Controlled Trials
Source: J Clin Med. 2025 Dec 19;15(1):2. doi: 10.3390/jcm15010002 (PMC12786405; doi:10.3390/jcm15010002)
Supplement: Supplementary file 1 [file jcm-15-00002-s001.zip › Table S2. Sensitivity analysis.pdf]

Table S2. Sensitivity analysis of main outcomes excluding one study at a time.

	BASIL-2,2023		BEST-CLI, 2022		Enzmann,2023		Totić,2020	
	OR(CI)	I2	OR(CI)	I2	OR(CI)	I2	OR(CI)	I2
**30-day mortality**	0.8 (0.34, 1.85)	0%	0.64 (0.17, 2.35)	12%	0.66 (0.34, 1.29)	0%	0.62 (0.31, 1.22)	0%
**Amputation**	1.51 (1.12, 2.04)	0%	1. 09 (0.63, 1.86)	13%	1.31 (0.80, 2.13)	49%	1.24 (0.87, 1.78)	28%
**Reintervention**	4.74 (3.63, 6.19)	0%	4.21 (2.63, 6.73)	0%	4.82 (3.65, 6.36)	0%	4.69 (3.64, 6.04)	0%
**Major reintervention**	2.53 (1.67, 3.84)	26%	2.6 (1.26, 5.37)	40%	3.19 (2.40, 4.24)	0%	2.78 (1.80, 4.29)	49%
**Tatol reintervention**	2.18 (1.78, 2.66)	0%	1.69 (1.07, 2.67)	22%	1.8 (1.20, 2.70)	50%	1.89 (1.35, 2.64)	50%
**MACE**	0.09 (0.05, 0.17)	0%	0.19 (0.09, 0.41)	0%	0.14 (0.07, 0.28)	33%	0.12 (0.06, 0.27)	44%
**Technical success**	0.09 (0.05, 0.16)	0%	0.17 (0.08, 0.35)	0%	0.12 (0.08, 0.20)	0%	0.11 (0.07, 0.18)	44%

MACE=Major adverse cardiovascular events, NA=not applicable
