# Supplementary figures and images for: Endovascular Treatment Versus Vein Bypass of Infrainguinal Peripheral Artery Disease: A Systematic Review and Meta-Analysis of Randomized Controlled Trials
Source: J Clin Med. 2025 Dec 19;15(1):2. doi: 10.3390/jcm15010002 (PMC12786405; doi:10.3390/jcm15010002)

Figure S3. Number needed to treat (NNT) and number needed to harm (NNH)

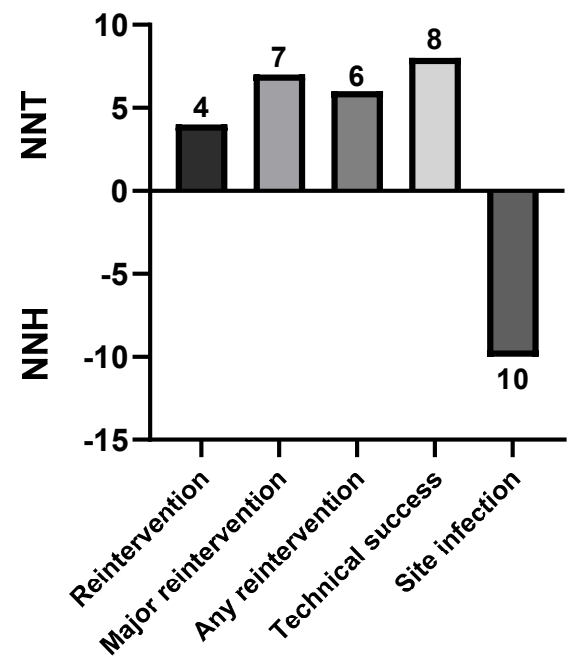

Supplement: Supplementary file 1 [file jcm-15-00002-s001.zip › Figure S3-NNT and NNH.pdf]
